# The S-Layer Glycome—Adding to the Sugar Coat of Bacteria

**DOI:** 10.1155/2011/127870

**Published:** 2010-08-10

**Authors:** Robin Ristl, Kerstin Steiner, Kristof Zarschler, Sonja Zayni, Paul Messner, Christina Schäffer

**Affiliations:** ^1^Department of NanoBiotechnology, Vienna Institute of BioTechnology, University of Natural Resources and Life Sciences, Muthgasse 11, 1190 Vienna, Austria; ^2^Centre for Biomolecular Sciences, University of St. Andrews, North Haugh, St. Andrews, Fife KY16 9ST, UK; ^3^Institute of Genetics, General Genetics, Dresden University of Technology, Zellescher Weg 20b, 01217 Dresden, Germany

## Abstract

The amazing repertoire of glycoconjugates present on bacterial cell surfaces includes lipopolysaccharides, capsular polysaccharides, lipooligosaccharides, exopolysaccharides, and glycoproteins. While the former are constituents of Gram-negative cells, we review here the cell surface S-layer glycoproteins of Gram-positive bacteria. S-layer glycoproteins have the unique feature of self-assembling into 2D lattices providing a display matrix for glycans with periodicity at the nanometer scale. Typically, bacterial S-layer glycans are O-glycosidically linked to serine, threonine, or tyrosine residues, and they rely on a much wider variety of constituents, glycosidic linkage types, and structures than their eukaryotic counterparts. As the S-layer glycome of several bacteria is unravelling, a picture of how S-layer glycoproteins are biosynthesized is evolving. X-ray crystallography experiments allowed first insights into the catalysis mechanism of selected enzymes. In the future, it will be exciting to fully exploit the S-layer glycome for glycoengineering purposes and to link it to the bacterial interactome.

## 1. Introduction

### 1.1. The Sweet Cell Surface of Bacteria at a Glance

Nature has equipped prokaryotic organisms from almost all phylogenetic branches with an amazing repertoire of components from its glycodiversification tool box, adding to the “sweetness” of their cell surface. The “sweetness” is derived from different kinds of polysaccharides, such as capsules or exopolysaccharides as well as glycoconjugates, such as lipopolysaccharides, lipooligosaccharides, and glycoproteins, all of which may additionally carry noncarbohydrate modifications. The complex type of biosynthesis of these prokaryotic carbohydrate components is truly amazing, and despite the tremendous progress in molecular biology it is still very difficult to see at present how the sequence of enzymatic reactions involved in the controlled biosynthesis of carbohydrate chains is regulated. Thus, it is reasonable to believe that the cellular sugar coat serves an important biological function. Prokaryotic glycoconjugates derive most of their structural diversity from the identities of their unusual sugar moieties. The addition of sugars to a nonglycosylated biomolecule changes its size and shape and this is likely to affect the access of proteolytic enzymes. Further, it will influence factors such as solubility, heat stability as well as many physical and chemical properties. Based on these properties, cell surface glycosylation may protect the prokaryotic cell from desiccation and other environmental stresses, contribute to the surface charge of the cell, facilitate adherence of bacteria to solid substrates or influence biofilm formation. Glycosylation of bacterial cell surfaces is furthermore emerging as a critical factor in symbiosis, pathogenesis, cell-cell interactions, and immune evasion. In addition, the presence of and the access to microbial glycoconjugates play a crucial role in the emerging field of biotechnology. For all of these reasons, it is desirable to fully understand the biochemical processes leading to the formation of prokaryotic glycoconjugates [1–4].

### 1.2. Bacterial S-Layer Glycoproteins

**Figure 1:**
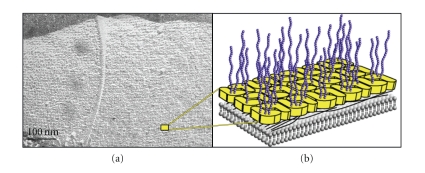
(a) Electron micrograph of the oblique S-layer glycoprotein lattice as observed on the cell surface of *G. stearothermophilus* NRS 2004/3a upon freeze-etching and platinum-carbon shadowing. Bar, 100 nm. (b) Inset, schematic representation of the cell wall illustrating the S-layer glycoproteins, with the S-layer glycan chains protruding into the exterior environment. Colour code: yellow S-layer protein; blue S-layer glycan chains; grey cytoplasmic membrane; black peptidoglycan.

Out of the components contributing to the cellular sugar coat, surface layer (S-layer) glycoproteins are a specific group. In the 1970s, S-layer glycoproteins were the first prokaryotic glycoproteins ever described [5, 6]. Since then, they have attracted considerable interest by the research community. This is not only because of their native potential for modification with a great variety of rare glycan structures (see below)—and, thus, representing ideal model systems for studying bacterial glycosylation—but also because of their unique self-assembly features. On the bacterial cell surface, but also *in vitro*, S-layer glycoproteins are characteristically aligned in 2D arrays, thereby periodically displaying the attached glycans to the ambient environment with nanometer-scaled accuracy (“nanolattice”) (Figure 1). High-resolution electron microscopy studies revealed that S-layer nanolattices can have oblique (p1, p2), square (p4), or hexagonal (p3, p6) symmetry [7].

S-layers, in general, are present as common outermost structures of the prokaryotic cell envelope. Molecular masses of S-layer proteins typically range from 40 to 170 kDa [7, 8], and glycosylation is the most frequent posttranslational modification these proteins undergo. Other covalent modifications include lipid attachment, phosphorylation, and methylation [9–11]. S-layer glycoproteins occur both in the domains of *Archaea* and *Bacteria. *While investigation of S-layer protein glycosylation of haloarchaea, which was initiated by the group of Strominger [5] and extended by Sumper and Wieland [12], is currently extensively being performed by the group of Eichler [2], our research focus is on the S-layer protein glycosylation of Gram-positive bacteria. There, the S-layer nanolattice assembles on the surface of the rigid peptidoglycan layer [13], to which it is attached via a “nonclassical” secondary cell wall polysaccharide [14, 15]. S-layer glycoprotein synthesis and assembly resembles a very efficient system. With an average bacterial generation time of ~20 minutes, at least 500 copies of a single glycoprotein species have to be synthesized per second, translocated to the cell surface, and incorporated into the existing S-layer nanolattice [7, 16, 17]. This corresponds to ~20% of the total protein synthesis effort of a bacterium being devoted to S-layer production. Recent data indicate that S-layer glycosylation lags behind protein biosynthesis [18].

## 2. Composition and Structure of Bacterial S-Layer Glycoproteins

### 2.1. Bacteria with S-Layer Glycosylation

Although no precise function could be attributed to bacterial S-layer glycoprotein glycans so far, an enormous biosynthesis effort is undertaken by various bacteria from different phylogenetic branches to glycosylate their S-layer proteins (see below). Thus, it is conceivable that glycosylation adds significantly to the potential functional spectrum of S-layer proteins [3, 12]. Besides Gram-positive bacteria and archaea, for which S-layer glycosylation has been confirmed by various research groups in the past, only recently there were first reports on Gram-negative bacteria possessing an S-layer glycoprotein layer; these are the pathogenic species *Tannerella forsythia* [19] and presumably *Parabacteroides distasonis* [20]. Linking S-layer protein glycosylation to pathogenicity is shedding new light into a potential *in vivo* function of S-layer glycans.

About 15 different S-layer glycoprotein glycan structures have been fully or partially elucidated so far, and there are currently more than 25 further reports on glycan modifications of S-layer proteins according to biochemical evidence (Table 1). The degree of glycosylation of S-layer proteins generally varies between 1% and 10% (w/w).

From the accumulated data it is evident that S-layer protein glycosylation cannot serve as a criterion for taxonomic typing at the genus level. However, S-layer glycans are useful tools for typing strains. It is interesting to note that the S-layer glycans present in fresh isolates may change or even be lost after prolonged cultivation of the bacteria in rich synthetic growth media. This supports the assumption that S-layer glycans provide a selection advantage for the bacteria in the competitive natural habitat.

### 2.2. Structural Repertoire of Bacterial S-Layer Glycans

Prokaryotic organisms are capable of forming both S-layer protein attached N- and O-glycans. While the former have so far been exclusively found in the archaeal domain [2], bacterial S-layer O-glycans from various members of the *Bacillaceae* family have been quite extensively studied during the past two decades in our laboratory [3, 63, 64]. 

**Figure 2:**
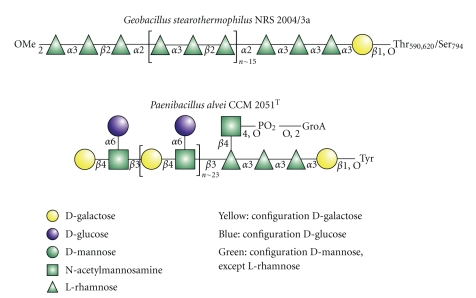
S-layer glycan structures of *G. stearothermophilus* NRS 2004/3a and *P. alvei* CCM 2051^T^. Symbols for representation of the glycan structures are according to the Consortium for Functional Glycomics (http://web.mit.edu/glycomics/consortium/whatsnew.shtml).

In addition to more common sugars present in eukaryotic glycans, such as *β*-D-Glc*p,* *α*-D-Gal*p*, *β*-D-Gal*p*, *β*-D-Gal*f,* *α*-D-Man*p,* *α*-D-Glc*p*NAc, *α*-D-Gal*p*NAc, and *β*-D-Gal*p*NAc, some S-layer glycans include rare sugars such as *α*-D-Rha*p*, *β*-L-Rha*p,* *α*-D-Fuc, *β*-D-Man*p*NAc, *α*-D-Fuc*p*NAc, *β*-D-Qui*p*NAc, or *β*-D-*glycero-*D-*manno*-Hep*p*, some of which are typical constituents of lipopolysaccharides (LPS) of Gram-negative bacteria [65]. Strain specifically, two to six of these sugars, build up the repeating units of the S-layer glycoprotein glycans. The repeating units may be linear (e.g., *Geobacillus stearothermophilus* NRS 2004/3a, Figure 2) or branched (e.g.,* Paenibacillus alvei *CCM 2051^T^, Figure 2) and are polymerized to various degrees (usually between 15 and 50 repeats) to comprise long-chain S-layer heterosaccharides [10, 66]. The Gaussian distribution of S-layer glycan chain lengths is a result of the different degrees of polymerization of individual repeating units and is reflected in the well-known phenomenon of glycan microheterogeneity. Additionally, some S-layer glycans are modified with noncarbohydrate substituents, such as phosphoglyceric acid [46], O-methyl-groups (2-O-Me, 3-O-Me) [34, 67], *α*-(*R*)-*N*-acetylmuramic acid, or *N-*acetylglucosamine [37]. Interestingly, the latter residues are present as capping elements of the terminal sugar residue at the nonreducing end of the respective S-layer glycan chain, which may imply their function as termination signal for chain elongation as was also reported for LPS O-antigen biosynthesis [65, 68]. Most of the investigated S-layer glycans are linked via a variable adaptor saccharide, which frequently contains *α*1,3-linked L-rhamnose residues, to specific O-glycosylation sites of the S-layer protein. Besides the amino acids serine and threonine, which are preserved as O-glycosylation sites through all domains of life, in S-layer proteins the chemically more stable O-glycosidic linkage to tyrosine is additionally present [48]. Identified carbohydrate-protein linkage types include *β*-Glc→Tyr [58, 61], *β*-Gal→Tyr [46, 55, 56], *β*-Gal→Thr/Ser [34], *β*-GalNAc→Thr/Ser [67, 69], and *α*-Glc → Ser [40]. Commonly, S-layer proteins are glycosylated at multiple sites, which may be either clustered or distributed over the whole protein. So far, between two and four glycosylation sites have been identified per individual S-layer protein. While the structure of the S-layer glycan is conserved for a given S-layer protein, there is obvious variation in the protein primary sequence surrounding the glycosylated amino acid. Currently it is under investigation, to which extent the different glycosylation sites are occupied in the mature glycoprotein. 

In most instances, the overall architecture of S-layer glycoprotein glycans resembles that of LPS of Gram-negative bacteria [70]. Either category of cell surface glycoconjugate possesses a tripartite scheme. This scheme comprises a glycan chain built of a variable number of repeating units that is linked via a variable adaptor saccharide to a specific compound of the bacterial cell wall, which is the S-layer protein moiety in the case of S-layer glycoproteins and lipid A-core in the case of LPS.

In the following (Section 3), the S-layer glycoprotein glycans of *G. stearothermophilus* NRS 2004/3a (Figure 2) and *P. alvei *CCM 2051^T^ (Figure 2) will be discussed as showcases to illustrate very recent data on the biosynthesis machinery governing S-layer protein glycosylation. The S-layer glycan of the thermophilic bacterium *G. stearothermophilus* NRS 2004/3a is a poly-L-rhamnan structure, consisting of 13 to 18 [→2)-*α*-L-Rha*p*-(1→3)-*β*-L-Rha*p*-(1→2)-*α*-L-Rha*p*-(1→] trisaccharide repeating units, which is O-glycosidically linked via an adaptor saccharide built of two to three *α*1,3-linked L-rhamnose residues and a *β*-D-Gal residue to the amino acids Thr_590_, Thr_620_, and Ser_794_ of the S-layer protein precursor SgsE [34, 71]. As a specific feature, the terminal rhamnose residue is 2-O*-*methylated. In the Gram-positive, mesophilic bacterium *P. alvei* CCM 2051^T^, a branched S-layer polysaccharide consisting of 22 to 25 [→3)-*β*-D-Gal*p*-(1[*α*-D-Glc*p*-(1→6)] →4)-*β*-D-Man*p*NAc-(1→] repeating units is O-glycosidically linked via an adaptor with the structure -[GroA-2→OPO_2_→4-*β*-D-Man*p*NAc-(1→4)]  →3)-*α*-L-Rha*p*-(1→3)-*α*-L-Rha*p*-(1→3)-*α*-L-Rha*p*-(1→3)-*β*-D-Gal*p*-(1→ to at least two specific tyrosine residues of the S-layer protein SpaA [46, 48].

Based on the p2 symmetry of the S-layer nanolattices of *G. stearothermophilus* NRS 2004/3a and *P. alvei* CCM 2051^T^ and assuming that each S-layer protein subunit is modified with two glycan chains, an average-sized cell of either bacterium displays a total number of ~7,000 glycan chains on the cell surface. This indicates that S-layer protein biosynthesis and export must be highly coordinated with S-layer glycan biosynthesis. Regulation mechanisms interlinking these events are still to be investigated.

## 3. Building S-Layer Glycans—Insights into the Biosynthesis Machinery behind S-Layer Glycosylation

### 3.1. The Genetic Basis for S-Layer Protein Glycosylation

For a long time, the understanding of the genetic basis for S-layer protein glycosylation was lagging behind the structural analyses, because of the lack of suitable molecular tools. An important milestone was accomplished with the identification of the first S-layer glycosylation (*slg*) gene cluster in the bacterium *G. stearothermophilus* NRS 2004/3a [18]. Over the past five years, several *slg* gene clusters have been identified, sequenced, and characterized. This is in agreement with the frequently encountered strategy of encoding bacterial polysaccharide biosynthesis routes (e.g., LPS O-antigens) by chromosomal operons or gene clusters [72]. While investigation of polysaccharide gene clusters in Gram-negative bacteria began some 30 years ago [73, 74], research on comparable gene clusters of Gram-positive bacteria (e.g., S-layer glycans, exopolysaccharides) has advanced only more recently.

#### 3.1.1. Identification of slg Gene Clusters

Currently, most data for *slg* gene clusters is available from the bacteria *G. stearothermophilus* NRS 2004/3a (GenBank AF328862) [18], *P. alvei* CCM 2051^T^ (GenBank HM011508) [47], *Geobacillus tepidamans* GS5-97^T^ (GenBank AY883421) [38] as well as from *Aneurinibacillus thermoaerophilus* strains L420-91^T^ (GenBank AY442352), and DSM 10155/G^+^ (GenBank AF324836) [18]. In addition, a partial *slg* gene cluster sequence is available from *Thermoanaerobacterium thermosaccharolyticum* E207-71 (GenBank AY422724) [18]. 

*Slg* gene clusters have been originally identified based on the presence of L-rhamnose in the respective S-layer glycans and the knowledge that the *rmlABCD* genes involved in biosynthesis of L-rhamnose are highly conserved among Gram-positive and Gram-negative bacteria [75]. Degenerate primers designed for motifs found in RmlA and RmlB allowed the detection of *rml *operons in the surveyed strains, which served as an entry point for identifying complete *slg* gene clusters by chromosome walking. All these *slg* gene clusters contain consecutively arranged genes for glycosyltransferases, glycan processing enzymes, and membrane transporter components. Interestingly, for biosynthesizing the complete S-layer glycans, these genes work in concert with house-keeping functions. For instance, in *G. stearothermophilus* NRS 2004/3a, genes for the biosynthesis of nucleotide-activated L-rhamnose (*rmlABCD*) are present, but the cluster does not code for the biosynthesis of the precursor of the galactose linkage sugar (UDP-Gal) [32]. Unlike the situation in *G. stearothermophilus* NRS 2004/3a, the *slg* locus of *P. alvei* CCM 2051^T^ includes genes for the biosynthesis of the nucleotide-activated S-layer glycan constituents glycerol, galactose, glucose, and L-rhamnose, but lacks genes coding for enzymes involved in the biosynthesis of nucleotide-activated *N-*acetylmannosamine, which is also part of the S-layer glycan repeating unit [48].

#### 3.1.2. Genetic Organization of slg Gene Clusters

For comparing *slg* gene clusters with other known bacterial polysaccharide gene clusters, several features can be taken into account, including the percentage G+C content, a characteristic order of genes, and the type of flanking genes [65, 76, 77].

**Figure 3:**
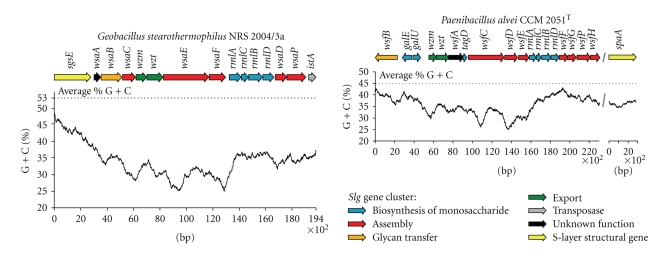
Schematic representation of the *slg* gene clusters of the two model bacteria *G. stearothermophilus* NRS 2004/3a and *P. alvei* CCM2051^T^. Individual stages of the S-layer glycosylation process are colour coded as follows: blue, monosaccharide biosynthesis; red, glycan assembly; orange, glycan transfer; green, export; grey, transposase; black, unknown function; yellow, S-layer protein. The corresponding percentage G+C base composition is given below each *slg* gene cluster map. Designation of ORFs in alphabetical order is as follows: *rmlA*, glucose-1-phosphate thymidylyltransferase; *rmlB*, dTDP-D-glucose 4,6-dehydratase; *rmlC*, dTDP-dehydrorhamnose 3,5-epimerase; *rmlD,* dTDP-dehydrorhamnose reductase; *sgsE, *S-layer protein precursor of *G. stearothermophilus* NRS 2004/3a; *spaA*, S-layer protein precursor of *P. alvei* CCM 2051^T^; *tagD*, putative glycerol-3-phosphate cytidyltransferase; *wsaA*, TPR-repeat containing protein; *wsaB*, putative oligosaccharyl:protein transferase; *wsaC*, *α*1,3-rhamnosyltransferase; *wsaD,* *α*1,3-rhamnosyltransferase; *wsaE*, *α*1,2-rhamnosyltransferase, *α*1,3-rhamnosyltransferase, 2-O-methyltransferase; *wsaF*, *β*1,2-rhamnosyltransferase; *wsaP*, UDP-Gal:phosphoryl-polyprenol Gal-1-phosphate transferase;* wsfA*, asparagine synthase homologue; *wsfC*, putative CDP-glycerol:poly(glycerophosphate) glycerophosphotransferase, putative *β*1,4-galactosyltransferase, putative *β*1,3-*N-*acetylmannosamine transferase; *wsfD*, putative *α*1,6-glucosyltransferase; *wsfE*, putative *β*1,4-*N-*acetylmannosamine transferase; *wsfF*, putative *α*1,3-rhamnosyltransferase; *wsfG*, putative *α*1,3-rhamnosyltransferase;* wsfH*, putative UDP-Glc:phosphoryl-polyprenol Glc-1-phosphate transferase; *wsfP*, UDP-Gal:phosphoryl-polyprenol Gal-1-phosphate transferase; *wzm*, ABC transporter integral membrane protein; *wzt, *ABC transporter nucleotide-binding protein.

Despite the low number of *slg* gene clusters that are available so far, some organizational principles can be inferred as follows: (i) glycosyltransferase genes responsible for the formation of the S-layer glycan repeating unit, such as *wsaE* and *wsaF* in *G. stearothermophilus* NRS 2004/3a (Figure 3) or *wsfC* and *wsfE* in *P. alvei* CCM 2051^T^ (Figure 3), are found in the central region of the *slg* gene clusters; (ii) the ABC-transporter genes *wzm *and *wzt* are located upstream of these genes, either immediately or separated by only one open reading frame (ORF); (iii) the *rmlABCD* genes and most other genes required for biosynthesis of specific nucleotide-activated glycoses are found downstream of the central region.

The G + C content of the genes encoded in the examined *slg* gene clusters is mostly between 30% and 43%, which, in each case, is lower than the average percentage G+C content of the residual genome of the respective bacterium. This is, for instance, 45% for *P. alvei* CCM 2051^T^ and 53% for *G. stearothermophilus* NRS 2004/3a [18, 48]. It is interesting to note that the genes in the central regions of *slg* gene clusters, which are the most strain specific ones, exhibit the lowest G + C contents. This compares to *cps* loci of *Streptococcus pneumoniae*, where serotype specific enzymes, especially *wzx* and *wzy*, are of lower G + C content, while the surrounding biosynthesis and regulatory genes within the cluster are of average G + C content [77]. In general, as has been also argued for LPS gene clusters, the low G + C content might indicate evolutionary acquirement of the glycosylation potential by lateral gene transfer [72].

Genes within the *slg* gene clusters are closely arranged, some have overlapping termination and initiation codons, like *wzm*, *wzt,* and *wsaE* in G*. stearothermophilus* NRS 2004/3a and *wsfF* and *wsfG* in *P. alvei* CCM 2051^T^ [32, 48]. A tight organization and overlap of ORFs has been reported in O-polysaccharide clusters, too [76], for instance, in the *rfb* locus of *S. enterica* C1 [78] and in the O-antigen biosynthesis locus of *Leptospira borgpetersenii* serovar Hardjobovis [79]. Concerning the mode of transcription of *slg* gene clusters, two different scenarios have been found, so far. In *G. stearothermophilus* NRS 2004/3a, the gene cluster is a polycistronic unit, with all genes being orientated in the same direction. While in *P. alvei* CCM 2051^T^, the UDP-galactose synthesis genes *galE* and *galU* as well as the putative oligosaccharyl: S-layer protein O-transferase (O-OTase) gene *wsfB* are transcribed monocistronically, in addition to the larger polycistronic part [48], which, in this bacterium, implies the presence of an “*slg *gene locus” rather than of an “*slg* gene cluster”. Regarding the chromosomal location of the *slg* gene clusters, no preferential insertion site on the chromosome could be identified, so far. Also no JUMPstart sequence that is located upstream of several O-polysaccharide gene clusters has been found [80].

The S-layer structural genes encoding the target proteins for glycosylation are transcribed monocistronically and independently of the *slg* gene cluster or locus [32, 81]. Whereas the S-layer structural genes *sgsE* and *sgtA* of *G. stearothermophilus* NRS 2004/3a and *G. tepidamans* GS5-97^T^, respectively, are located in close vicinity to the corresponding *slg* gene clusters [32, 38], this is not the case for *P. alvei* CCM 2051^T^ and different *Aneurinibacillus thermoaerophilus* strains, where a more distant chromosomal location has been reported [18, 48]. 

Despite the overall number of known *slg* gene clusters or loci is still too low for generalization, the principal organization compares well with other bacterial polysaccharide gene clusters. For instance, a survey on the exopolysaccharide (*eps*) gene clusters from diverse lactic acid bacteria revealed that they have an operon structure with a high coding density. The genes are orientated in one direction and are transcribed as a single RNA [82]. The sequence of the functions of the genes in all of the investigated *eps *gene clusters was found to be as follows: regulation, chain length determination, biosynthesis of the repeating unit, and export.

It is conceivable to assume that the principal potential for S-layer protein glycosylation is obviously more widespread among bacteria than was initially assumed. This can be deduced from the identification of catalytically active Rml enzymes in *G. stearothermophilus* strains with an S-layer glycosylation negative phenotype and devoid of other rhamnose-containing glycoconjugates [32]. The corresponding typical and active *rml* operons were found to be flanked by putative glycosyltransferases, which may be indicative of a kind of silent glycosylation gene cluster.

### 3.2. Proposal of a Biosynthesis Pathway for S-Layer Glycans

Over the past five years, major strides have been made in identifying bacterial genes whose products are involved in the S-layer protein O-glycosylation process. 

**Figure 4:**
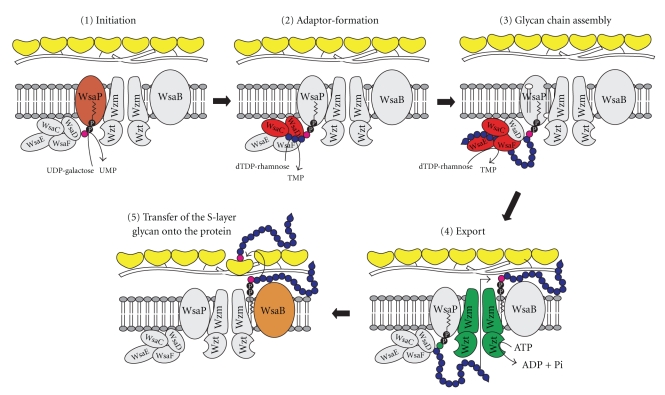
Proposed model of S-layer glycoprotein glycan biosynthesis in *G. stearothermophilus *NRS 2004/3a. (1) Initiation: transfer of a Gal residue from UDP-*α*-D-Gal to a lipid carrier catalyzed by WsaP. (2) Adaptor formation: *α*1,3-linkage of a rhamnose residue from dTDP-*β*-L-Rha to the primer by the action of WsaD, followed by the transfer of one or two additional *α*1,3-linked rhamnoses by the action of WsaC. (3) Glycan chain assembly: formation of repeating unit-like structures by action of the rhamnosyltransferases WsaE and WsaF, whereby WsaE is forming the *α*1,2- and the *α*1,3-linkages, and WsaF is forming the *α*1,2-linkage. Shown is termination of chain growth by 2-O-methylation of the terminal repeating unit, catalyzed by the O-methyltransferase domain of WsaE. (4) Export: the Wzt component of the Wzm/Wzt ABC transporter system is predicted to be responsible for binding of the 2-O-methylated glycan chain and its subsequent export through the membrane. (5) Transfer of the S-layer glycan onto the protein: the final transfer of the completed S-layer glycan to the S-layer protein would be catalyzed by the oligosaccharyltransferase WsaB. This research was originally published in the *Journal of Biological Chemistry *[35]. The American Society for Biochemistry and Molecular Biology. For colour code see Figure 3; pink, linkage glycose.

For our research effort, *G. stearothermophilus* NRS 2004/3a and *P. alvei* CCM 2051^T^ are serving as model organisms (compare with Figure 2). In *G. stearothermophilus* NRS 2004/3a, several genes from the S-layer glycosylation machinery have been cloned and their products functionally characterized by combined biochemical, NMR, and MS approaches [35]. For *P. alvei* CCM 2051^T^, mutants with deletions in specific genes from the S-layer glycosylation locus were constructed and the effects on the S-layer glycosylation patterns were monitored by MS [47, 48]. Either approach led to a proposal of a complete biosynthesis pathway of the S-layer glycoproteins. The combined picture resulting from those data is explained below, addressing the different biosynthesis stages of S-layer glycoprotein glycans—initiation, assembly, and transfer onto the protein—separately. A pictorial model of S-layer glycoprotein biosynthesis is given in Figure 4, exemplified with *G. stearothermophilus* NRS 2004/3a.

#### 3.2.1. Initiation

The initiation enzymes of S-layer glycoprotein glycan biosynthesis in *G. stearothermophilus* NRS 2004/3a and *P. alvei* CCM 2051^T^ are the UDP-Gal:phosphoryl-polyprenol Gal-1-phosphate-transferases WsaP, and WsfP, respectively. WsaP was shown to transfer Gal-1-P from UDP-Gal to phosphoryl-polyprenol *in vitro* and to reconstitute the function of WbaP in WbaP-deficient strains of *Escherichia coli* and *Salmonella enterica* [83]. Deletion of *wsfP* in *P. alvei* CCM 2051^T^ resulted in loss of S-layer glycosylation in this strain, while the original phenotype could be restored by expression of either WsfP or WsaP [47]. WsaP and WsfP share 60% identity and 75% similarity. They have been readily identified in the respective *slg* gene cluster, because both enzymes show high sequence homology to members of the poly-isopropyl-phosphate hexose-1-phosphate transferase (PHPT) family such as WbaP [84]. Either enzyme from the S-layer glycosylation pathway exhibits a distinct topology comprising five transmembrane domains and a large cytoplasmic C-terminus characteristic of PHPTs [18, 47, 83]. For WsaP, the functional domain was localized at the C-terminus of the enzyme [83], which is in agreement with the data presented for WbaP [85].

The involvement of WbaP homologues in the S-layer glycosylation pathway of *G. stearothermophilus* NRS 2004/3a and *P. alvei* CCM 2051^T^ was rather unexpected, since in the corresponding *slg* gene clusters, the presence of the ABC-transporter constituents Wzm and Wzt is predicted. Consequently, in analogy to what is known from the well-investigated routes of LPS O-antigen biosynthesis [65], the initiation of S-layer glycan assembly should be catalyzed by a WecA homologue, which is a GlcNAc-1-phosphate transferase [65].

#### 3.2.2. Assembly

S-layer glycans in *G. stearothermophilus* NRS 2004/3a and *P. alvei* CCM 2051^T^ are assembled stepwise by the consecutive transfer of glycose residues from the nucleotide-activated forms to the lipid-bound growing glycan chain, with the individual transfer reactions being catalyzed by specific glycosyltransferases [35, 48]. This scheme implies glycan chain growth from the nonreducing end and, thus, resembles the ABC-transporter-dependent pathway of O-polysaccharide biosynthesis [65].

Assembly of the poly-L-rhamnan S-layer glycan of *G. stearothermophilus* NRS 2004/3a (compare with Figure 2) has been studied in different *in vitro* set-ups [35]. The *slg* gene cluster of this bacterium (Figure 3) encodes four rhamnosyltransferases, named WsaC through WsaF, which are involved in the biosynthesis of the S-layer glycan. The individual reactions catalyzed by each of the four enzymes have been elucidated in detail by their respective ability to transfer rhamnose from dTDP-*β*-L-Rha to different synthetic substrates resembling the different stages of the lipid-bound growing glycan chain [35]. Membrane-anchored WsaD was found to catalyze the initial rhamnosylation reaction which is the transfer of activated *β*-L-Rha to the lipid-bound galactose primer (compare with Section 3.2.1). Subsequently, WsaC adds one or two additional L-rhamnose residues, thereby completing the adaptor saccharide. The repeating units (individual repeating units can only be distinguished by the different types of glycosidic linkages between the rhamnose residues) are synthesized in a concerted action of the soluble proteins WsaE and WsaF. WsaF catalyzes the *β*1,2-linkage, while WsaE is a rather unusual trifunctional enzyme. It contains three active domains, two of which are glycosyltransferase domains responsible for the formation of the *α*1,3- and *α*1,2-linkage of the glycan repeating unit. N-terminally, a methyltransferase domain is located which was found to methylate an *α*1,3-linked rhamnose, indicating its role in the formation of the 2-O-methyl cap, and, hence, in termination of S-layer glycan chain elongation as discussed for the O8 and O9 antigens of *E. coli* [86]. The methylation reaction catalyzed by WsaE was shown to be independent of chain length *in vitro.* Consequently, an additional factor for the determination of glycan length is possibly required *in vivo*.

Insight into the assembly of the S-layer glycan of *P. alvei* CCM 2051^T^ was gained through a systematic gene deletion approach of the individual genes encoded by the *slg* gene cluster of this bacterium [48] (compare with Figures 2 and 3). The biosynthesis pathway that was proposed based on the detailed analysis of the glycosylation pattern of the individual mutants corroborates the scenario described for *G. stearothermophilus* NRS 2004/3a, albeit additional information as to how a branched S-layer glycan may be created is unravelled. The first building block of the *P. alvei* CCM 2051^T^ S-layer glycan is the adaptor saccharide with the structure -[GroA-2→OPO_2_→4-*β*-D-Man*p*NAc-(1→4)] →3)-*α*-L-Rha*p*-(1→3)-*α*-L-Rha*p*-(1→3)-*α*-L-Rha*p*-(1→3)-*β*-D-Gal*p*-(1→] [46]. WsfG transfers the first L-rhamnose residue to the initial lipid-linked galactose obtained upon catalysis of WsfP (see Section 3.2.1). Subsequently, WsfF adds two more L-rhamnose residues. Both enzymes contain predicted transmembrane domains. The branching ManNAc residue is predicted to be transferred by WsfE, and the GroA-2-phosphate is likely to be added by the CDP-glycerol:poly(glycerophosphate) glycerophosphotransferase domain of WsfC. WsfC contains two more active domains, most probably catalyzing the alternating transfer of D-galactose and D-ManNAc, thus synthesizing the repeating unit backbone [→3)-*β*-D-Gal*p*-(1→4)-*β*-D-Man*p*NAc-(1→] of the S-layer glycan [45]. A branching glucose would be *α*1,6-linked to the repeating unit ManNAc residue via a separate mechanism that was shown to depend on the activity of WsfH and WsfD. According to our current interpretation, WsfH would transfer a single glucose residue from UDP-Glc to a separate lipid carrier, from which it would be further transferred to the S-layer glycan by the activity of WsfD. A comparable system is found for different *E. coli* serotypes [87] and in phage-encoded O-antigen modification systems [88]. There, an additional flippase is involved as link between cytoplasmically generated undecaprenyl phosphate-bound glucose and transfer to the periplasmic space. The absence of a flippase in the *slg* gene cluster might indicate that in *P. alvei* CCM 2051^T^, the transfer of the branching glucose occurs on the cytoplasmic side of the cytoplasmic membrane. However, an alternative transport mechanism for lipid-linked glucose cannot be ruled out.

#### 3.2.3. Transfer of the S-Layer Glycan onto the Protein

The *slg* gene clusters from both *G. stearothermophilus* NRS 2004/3a and *P. alvei* CCM 2051^T^ contain the integral membrane protein Wzm and the nucleotide-binding protein Wzt constituting an ABC transporter [89–91]. In analogy to the ABC-dependent pathway of O-polysaccharide synthesis, the ABC transporter would be responsible for the transport of complete lipid-bound S-layer glycans across the cytoplasmic membrane. Currently, it is being investigated, if the ABC transporters involved in S-layer glycan export have a certain chain-length preference and, if present, how it is specified [68].

The final protein glycosylation step, which is the transfer of the elongated glycan chain onto the S-layer protein, would most likely occur cosecretionally. It would be catalyzed by a specific *O*-OTase. The glycosylation sites on the *G. stearothermophilus* NRS 2004/3a S-layer protein precursor are the amino acids Thr_590_, Thr_620_, and Ser_794_ [34, 71], while in *P. alvei* CCM 2051^T^, at least two tyrosine residues are found to be glycosylated [46]. Interest in bacterial OTases has been rising over the past few years and much insight has been gained from studies on the pilin *O*-glycosyltransferases PilO of *Pseudomonas aeruginosa* and PglL of *Neisseria meningitidis* [92, 93] as well as on PglB of the *N*-glycosylation system of *Campylobacter jejuni* [94, 95]. The predicted *O*-OTases of *G. stearothermophilus* NRS 2004/3a and *P. alvei* CCM 2051^T^, WsaB and WsfB, respectively, both contain the *O*-antigen ligase domain Wzy_C, which is found in both PilO and PglL [96]. WsfB and PglL contain a C-terminally located tetratricopeptide (TPR) motif, which is described as mediator of protein-protein interactions [97–99]. While WsaB does not include a TPR motif, the ORF of *wsaA* located just upstream of *wsaB* encodes a 168-amino acid protein of otherwise unassigned function that contains a TPR motif. If WsaA in some way complements WsaB for the lack of a TPR motif is currently under investigation. Eventually, the signal sequence would be cleaved from the S-layer protein portion, and the mature S-layer glycoprotein would be deemed to 2D crystallization on the bacterial cell surface to form the S-layer glycoprotein nanolattice.

Summarizing, S-layer glycan biosyntheses in *G. stearothermophilus* NRS 2004/3a and *P. alvei* CCM 2051^T^ follow similar pathways. The initiation enzymes are highly homologous Gal-1-phosphate transferases; the adaptor region of either glycan is synthesized by two membrane-bound glycosyltransferases. In both bacteria, a large cytoplasmic multidomain enzyme is involved in the formation of the repeating unit and has an additional function in catalyzing a further glycan modification (i.e., methylation and addition of glyceric acid phosphate, resp.). Multidomain enzymes are also found in O-polysaccharide synthesis pathways; an example is the multidomain mannosyltransferase WbdA found in *E. coli* serotypes O8 and O9a [100, 101]. Export across the cytoplasmic membrane involving a dedicated ABC transporter-dependent pathway and transfer of the completed S-layer glycan chain onto the protein *O*-glycosylation sites would follow identical routes. From this scenario it is obvious that different modules from the well-known LPS O-antigen biosynthesis pathways are used for S-layer glycoprotein glycan biosynthesis, albeit in new combination. This is clearly inferred from the involvement of a WbaP homologue, which is otherwise typical of the ABC transporter-independent pathway of O-polysaccharide biosynthesis [65].

## 4. Structure—Function Relationship of Selected Enzymes from S-Layer Glycosylation

During the past three years, several enzymes from S-layer glycosylation pathways have been crystallized, which allows, for the first time, insight into their reaction mechanisms. 

### 4.1. Crystal Structure of the dTDP-4-Keto-6-Deoxy-D-Glucose-3,4-Ketoisomerase FdtA

The repeating unit of the glycan chain of the S-layer of the bacterium *A. thermoaerophilus* L420-91^T^ is a hexasaccharide composed of four *α*-D-rhamnose residues and two 3-acetamido-3,6-dideoxy-*α*-D-galactose (Fuc*p*3NAc) residues. The precursor of the latter component, dTDP-Fuc*p*3NAc, is synthesized by the action of five enzymes from *α*-D-glucose-1-phosphate [102]. A crucial step in the biosynthesis is the conversion of the 4-keto substrate dTDP-4-keto-6-deoxyglucose into the 3-keto product dTDP-3-keto-6-deoxygalactose with accompanying inversion of the configuration about C-4′ of the hexose ring. This step is catalyzed by the isomerase FdtA [102].

**Figure 5:**
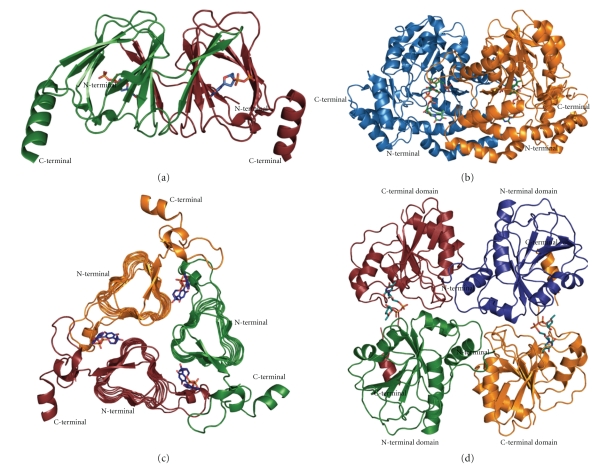
Structure of a (a) dimer of FdtA in complex with dTDP (2PA7), (b) dimer of QdtB in complex with pyridoxal 5′ phosphates (PLP) and dTDP-3-keto-6-deoxy-D-glucose (3FRK), (c) trimer of QdtC in complex with CoA and dTDP-D-Qui*p*3N (3FSB), (d) dimer of WsaF in complex with dTDP-L-Rha (2X0F). The individual subunits are colour-coded in (a) red and green, (b) blue and orange, and (c) red, green, and orange, in (d), in addition to the subunits, the domains are colour coded as follows: subunit 1: green/red, subunit 2: blue/orange. The protein chains are depicted as cartoon presentation. The ligands in all structures are shown as sticks. The figures were depicted using PyMOL (DeLano Scientific).

The structure of FdtA was solved in complex with dTDP to a nominal resolution of 1.5 Å by Davis and coworkers [103]. The FdtA dimer has an almost jellyfish-like appearance with the sole *α*-helices representing the tentacles (Figure 5(a)). The overall structure of each subunit adopts a *β*-barrel motif formed by two layers of antiparallel *β*-sheets consisting of five *β*-strands each. The dimer is formed by domain swapping whereby *β*-strands two and three from one subunit form part of a *β*-sheet in the second subunit. The active site architecture of FdtA is characterized by a cluster of three histidine residues (His_49_, His_51_, and His_95_). His_49_ and His_51_ are strictly conserved among putative sugar isomerases in the database. The importance of these residues was further confirmed by site-directed mutagenesis and enzymatic assays. On the basis of the location of dTDP in the complex with FdtA, the substrate dTDP-4-keto-6-deoxyglucose was modelled into the active site cleft. This model places His_49_ and His_51_ near the hexose C-3′ oxygen and His_95_ near the hexose C-4′ oxygen. In the proposed mechanism, His_49_ would act as base by removing the hydrogen from the sugar C-3′ and shuttle it to the sugar C-4′ on the same side of the glycosyl group resulting in inversion of the configuration at C-4′. His_51_ might be involved in shuttling protons between the C-3′ and C-4′ oxygens. Sequence comparison with sugar isomerases revealed that enzymes that retain the configuration at C-4′ contain an arginine, and inverting isomerases contain a histidine at the position of His_95_ in FdtA. Overall, FdtA was the first structure in a new family of inverting sugar isomerases [103].

### 4.2. Crystal Structure of the Aminotransferase QdtB

3-acetamido-3,6-dideoxy-*α*-D-glucose (Qui*p*3NAc) is an unusual sugar found in the O-antigens of some Gram-negative bacteria and in the S-layer glycoprotein glycans of Gram-positive bacteria, for example,* Thermoanaerobacterium thermosaccharolyticum *E207-71 [60]. It is synthesized as dTDP-linked sugar by five different enzymes starting from glucose 1-phosphate [104]. In step four, QdtB aminates dTDP-3-keto-6-deoxy-D-glucose at the C-3′ position of the hexose. The structure of QdtB was solved recently to 2.15 Å resolution [105]. QdtB is a dimer and shows homology to the aspartate aminotransferase superfamily. Each subunit contains ten *β*-strands, seven of which form a mostly parallel *β*-sheet and the other three fold into an antiparallel *β*-sheet (Figure 5(b)). These *β*-sheets are surrounded by eleven *α*-helices. The catalytic site is located between the two domains with the residues Tyr_183_, His_309_ and Tyr_310_ from one subunit, and Lys_219_ from the other subunit interacting with dTDP-3-keto-6-deoxy-D-glucose. A reaction intermediate could be trapped within the active site cleft in the structure, the Schiff base between C-4′ of pyridoxal 5′ phosphates (PLP) and the amino nitrogen of the sugar. Furthermore, the structure revealed a lack of a specific interaction between the protein and C-4′ hydroxyl group of the hexose, which could lead to relaxed substrate specificity. This was further confirmed by the ability of QdtB to aminate dTDP-3-keto-6-deoxy-D-galactose [105].

### 4.3. Crystal Structure of the N-Acetyltransferase QdtC

QdtC is a CoA-dependent *N-*acetyltransferase that catalyzes the last step in the Qui*p*3NAc biosynthesis [104]. Interestingly, it can acetylate either dTDP-D-Qui*p*3N or dTDP-D-Fuc*p*3N. The structure of QdtC was determined in complex with CoA and either dTDP-D-Qui*p*3N or dTDP-D-Fuc*p*3N [106]. QdtC forms a physiological trimer. Each subunit interacts with both other subunits to form the trimer (Figure 5(c)). Each subunit consists of 32 *β*-strands that form a left-handed *β*-helix motif with 11 turns which is disrupted by two extended loops. The first one contains an *α*-helix and the second is involved in acetyl-CoA binding. The three active sites are located at the subunit-subunit interfaces. The acetyl-CoA binding sites are located between two subunits at one end of the trimer. Its phosphoryl groups point outwards toward the solvent, whereas the adenine ring, the pantothenate, and *β*-mercaptoethylamine are buried within the QdtC trimer. The adenine group of CoA interacts with Ala_99_ and the pantothenate with Ala_180_ and Gly_198_ from one subunit, respectively, whereas the 2-hydroxyl and the phosphoryl group of CoA interact with Thr_204_ and Lys_205_, respectively, and the *β*-mercaptoethylamine forms a hydrogen bond with Asp_160_ from the second subunit. Two subunits also contribute to the binding of the dTDP-sugars. Thr_117_ from one subunit forms a hydrogen bond to the O3 of 2-deoxyribose, whereas Tyr_86_ from the neighbouring subunit interacts with N3 of the thymidine ring. The pyrophosphoryl group forms interactions with one arginine from each of the subunits. The amino acid residues Glu_141_, Asn_159_, and Asp_160_ from one subunit and His_134_ from another subunit are responsible for anchoring the hexose moieties of the dTDP-sugars to the protein. Interestingly, the catalytic site shows only a slight movement in response to the different configuration at the C-4′-hydroxyl of dTDP-D-Qui*p*3N and dTDP-D-Fuc*p*3N. 

Overall, QdtC shows structural homology to PglD, an *N-*acetyltransferase, but the nucleotide-linked sugars are accommodated differently in the active site [107, 108]. Although in PglD, His_125_, which is also conserved in His_123_ of QdtC, was described as general base in the reaction mechanism, site-directed mutagenesis of His_123_ and several other potential candidates, including His_134_, Glu_141_, Asp_160_, and Asn_159_, revealed only reduced activity. This is more likely the result from reduced hexose binding than from the loss of an enzymatic base. Thus, a new reaction mechanism was proposed by Thoden and coworkers [106], which does not involve a general base provided by the protein. dTDP-D-Qui*p*3N would bind to the active site in an unprotonated form. One of the hydrogens on the sugar amino group is directed at the carboxamide group of Asn_159_, whereas the other is directed at the sulfur of acetyl-CoA. The lone pair of electrons on the nitrogens is directed to the carbonyl carbon of actyl-CoA. During the nucleophilic attack of the amino nitrogen, the bond between the carbonyl carbon and the sulfur of acetyl-CoA becomes longer and eventually breaks. Finally, the sulfur accepts a proton from the sugar amino group and by this serves as the catalytic base [106].

### 4.4. Crystal Structure of the Rhamnosyltransferase WsaF

**Figure 6:**
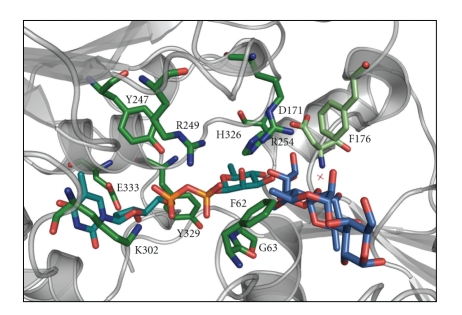
Close-up view of WsaF with dTDP-*β*-L-Rha bound in the binding pocket and tri-rhamnose galactose modelled into the putative acceptor binding tunnel. Colour code: turquoise, dTDP-Rha; blue, tri-rhamnose galactose.

The S-layer glycoprotein glycan from *G. stearothermophilus *NRS 2004/3a is mainly composed of repeating units of three rhamnose sugars linked by *α*1,3-, *α*1,2-, and *β*1,2-linkages (compare with Figure 2). The formation of the *β*1,2-linkage is catalyzed by the rhamnosyltransferase WsaF [35]. Recently, the structure of WsaF could be solved to a resolution of 2.3 Å [109, 110]. The structure of WsaF shows a dimer with homology to the glycosyltransferase (GT) 4-family in the CAZy (Carbohydrate-Active EnZYme) database [111]. Each subunit consists of two domains with the typical GT-B-fold of two Rossmann-fold domains (*β*/*α*/*β*) and a cleft between the two domains, which includes the catalytic centre (Figure 5(d)). The N-terminal domain presumably binds the acceptor (the growing rhamnan chain) and the C-terminal domain binds the substrate (dTDP-*β*-L-rhamnose). The structure of WsaF bound to dTDP and dTDP-*β*-L-rhamnose, coupled to side-directed mutagenesis and biochemical analysis, identified the residues that underlie catalysis and substrate recognition. The dTDP forms interactions with Arg_249_, Lys_302_, and Tyr_247_, all of which have been shown by mutagenesis to be important or critical for enzyme function possibly by stabilizing the pyrophosphate leaving group. Arg_254_ is placed immediately adjacent to the site of glycosidic bond formation, where it could interact with both donor and substrate sugars as well as facilitate catalysis. Glu_333_ of WsaF binds to the O3 of 2-deoxyribose and corresponds to the second Glu of an EX7E motif found in the active site of all structures of the GT4-family. However, instead of the first Glu, which is interacting with the hexose moiety in the other members, a proline residue is found in WsaF. Pro_325_ is part of an unusual proline-rich motif, PHPSYPPLE, where the central tyrosine points towards the likely location of rhamnose. The motif is conserved among sequence homologues of WsaF in BLAST, including some putative rhamnosyltransferases and may be a characteristic signature for rhamnose usage. It should be noted that WsaF is the first structure of a rhamnosyltransferase published so far. The N-terminal domain is likely to function as the acceptor-binding region but is not conserved among the known structures of the GT4 family due to the very different nature of the acceptors. A model of the acceptor places it in a tunnel that is lined by Asp_171_ and Phe_176_ with the C-2′ of the acceptor rhamnose close to dTDP-Rha (Figure 6). However, the catalytic mechanism of WsaF, like in many other retaining glycosyltransferases, is still controversial, because of the absence of an amino acid that could serve as nucleophile in the classic double-displacement mechanism.

## 5. Concluding Remarks

Glycosylation of proteins accounts for much of the biological diversity generated at the proteome level. In this review, we focus on a special type of glycoproteins present on a wide variety of bacterial cell surfaces, namely on S-layer glycoproteins. Our model bacteria are members of the *Bacillaceae* family that are covered with a 2D crystalline nanolattice composed of individual S-layer O-glycoproteins and displaying long-chain S-layer glycans with nanometer-scale periodicity. Structure- and compositionwise, S-layer glycans are amazingly diverse and differ distinctly from eukaryotic glycoproteins. From the detailed comparison of the S-layer glycoprotein glycan biosynthesis pathways in *G. stearothermophilus* NRS 2004/3a and in *P. alvei* CCM 2051^T^, it can be concluded that these pathways are based on different modules known from established bacterial polysaccharide biosynthesis routes, but they are utilized in new combination.

Despite recent advances in deciphering the bacterial S-layer glycome, leading to the proposal of a complete biosynthesis pathway for bacterial S-layer O-glycoprotein glycans, many questions remain still open. In addition to identifying and functionally characterizing glycosyltransferases, especially branching enzymes and multidomain enzymes that are frequently encountered during S-layer protein glycosylation, the requirements for translocation of the S-layer glycan chains across the cytoplasmic membrane and the final event of glycan transfer onto the S-layer protein need to be defined. Particular efforts will also specifically address the details about tyrosine O-glycosylation, which is frequently found in S-layer glycoproteins. In the future, it will be exciting to fully exploit the S-layer glycome for glycoengineering purposes and to link it to the bacterial interactome.

Carbohydrate-active enzymes from S-layer protein glycosylation pathways, especially those from nucleotide sugar biosynthesis, offer interesting options for therapeutic intervention. For instance, the immediate source for L-rhamnose, a common component of the cell wall and the capsule of many pathogenic bacteria as well as of many S-layer glycans, is dTDP-L-Rha. Since to date, neither rhamnose nor the RmlABCD genes responsible for dTDP-L-Rha biosynthesis have been identified in humans, these enzymes are ideal targets for inhibiting rhamnose-mediated bacterial pathogenicity [75].

Other enzymes from the S-layer glycome can be beneficial for synthesis purposes because of their unusual specificities or thermostability. Thermostable Rml enzymes from *A. thermoaerophilus* DSM 10155 allow higher productivity of dTDP-L-Rha [112]. Other enzymes such as an isomerase derived from *A. thermoaerophilus* DSM 420-91^T^ capable of synthesizing dTDP-3-keto-6-deoxygalactose from dTDP-4-keto-6-deoxyglucose [102] may be relevant for the synthesis of antibiotics that contain C-3-aminated deoxy-sugars, such as erythromycin or tylosin.

Since the S-layer glycome is connected with a molecular self-assembly system, our approach has a strong link to the field of nanobiotechnology, because means for organizing materials, such as biologically functional glycans, at the nanometer level are prime candidates for the fabrication of supramolecular structures and devices [113]. Nanobiotechnology applications of such devices that have additionally been tailored by glycoengineering may include the fields of receptor mimics, vaccine design, or drug delivery using carbohydrate recognition.

## Figures and Tables

**Table 1:** Overview on S-layer glycoprotein-covered bacteria.

Organism	Gene designation/Accession number	Lattice symmetry^a^/Lattice dimensions (nm)/Molecular mass (kD)	Biochemical evidence	Reference
*Aneurinibacillus thermoaerophilus,* strain L420-91^T^	*satA*/AY395578 *slg* cluster/AY442352	S/10.0/116	O-glycan structure Linkage region	[3, 18, 21]
*Aneurinibacillus thermoaerophilus *DSM 10155/G^+^	*satB*/AY395579	S/10.0/153	O-glycan structure Linkage region	[18, 22, 23]
*Bacillus smithii*, several strains	−/−	O /~11.2 and 10.3/132–138	Detection of glycoses	[24]
*Bacillus sp*., several strains	−/−	O,S,H/5–23/66–255	SDS-PAGE^b^, PAS^c^ staining	[25]
*Bacillus thuringiensis*, several strains	*c* *r* *y*020/−	−/−/65–130	SDS-PAGE^b^, PAS^c^ staining	[26]
*Campylobacter rectus, * strain ATCC 33238	*slp*/AB001876	H/−/150	Western blot	[27]
*Deinococcus radiodurans*, strain Sark	*HPI gene*/M17895	H/18/107	SDS-PAGE^b^, PAS^c^ staining, Monosaccharide analysis	[28, 29]
*Deinococcus radiodurans*, strain R1	*hpi*/DR2508, *slpA*/DR1185	H/−/99(37)	SDS-PAGE^b^, PAS^c^ staining	[30]
*Geobacillus stearothermophilus, * strain ATCC 12980^T^	*sbsC*/AF055578 dTDP-L-Rha operon/AY278519	O/9.5 and 5.2/122	Detection of L-rhamnose	[31, 32]
*Geobacillus stearothermophilus* ATCC 12980 variant	*sbsD*/AF228338	O/−/92^d^–175	SDS-PAGE^b^, PAS^c^ staining, Monosaccharide analysis	[33]
*Geobacillus stearothermophilus*, strain NRS 2004/3a	*sgs* /AF328862 *slg* cluster/AF328862	O/11.6 and 9.4/93^d^ –170	O-glycan structure Linkage region Biosynthesis	[32, 34, 35]
*Geobacillus stearothermophilus*, strain L32-65	*sgsF*/DQ414249 dTDP-L-Rha operon/AY278518	O/11.9 and 8.6/96	Detection of L-rhamnose	[32]
*Geobacillus tepidamans*, strain GS5-97^T^	*sgtA*/AY883421 *slg* cluster/ AY883421	O/11.2 and 7.9/106–166	O-glycan structure Linkage region	[36–38]
*Hyphomonas jannaschiana, *strain ATCC 33884	−/−	−/−/29 + 116	SDS- PAGE^b^, PAS^c^ staining	[39]
*Lactobacillus buchneri*, strain 41021/251	−/−	O/6.1 and 5.4/53	O-glycan structureLinkage region	[40]
*Lactobacillus helveticus, * strain ATCC 12046	−/−	O/9.6 and 4.5/43.5	SDS-PAGE^b^, PAS^c^ staining	[41]
*Lactobacillus kefir* JCM 5818 and several other strains	−/−	−/−/69	SDS- PAGE^b^, PAS^c^ staining Mass spectrometry	[42, 43]
*Methylomicrobium alcaliphilum*	−/−	H/36/10–45	SDS- PAGE^b^ Monosaccharide analysis	[44]
*Paenibacillus alvei,* strain CCM 2051^T^	*spaA*/FJ751775	O/10.0 and 7.9/240, 160, 105^d^	O-glycan structure Linkage region Biosynthesis	[45–48]
*Parabacteroides distasonis*	DgpA, DgpC/−	−/−/140, 90	SDS-PAGE^b^, PAS^c^ staining Monosaccharide analysis	[20]
*Sulfobacillus thermosulfidooxidans*	−/−	−/−/−	Monosaccharide analysis	[49]
*Synechococcus* sp., strain WH8102	*swmA*/AF056046	O/12/−	SDS- PAGE^b^, PAS^c^ staining	[50, 51]
*Tannerella forsythia, * strain ATCC 43037	*tfsA* + *tfsB*/AY423857	−/−/200 + 210	SDS- PAGE^b^, PAS^c^ staining	[19, 52, 53]
*Thermoanaerobacter kivui,* strain DSM 2030	*slp*/M31069	H/19 /~90	SDS- PAGE^b^, Deglycosylation	[54]
*Thermoanaerobacter thermohydrosulfuricus*, strain L111-69	*surA*/AJ401026 *sttA*/FJ656210	H/13.9/130	O-glycan structure Linkage region	[55, 56]
*Thermoanaerobacter thermohydrosulfuricus*, strain S102-70	−/−	H/16.3/94	O-glycan structure Linkage region	[57, 58]
*Thermoanaerobacter thermohydrosulfuricus*, strains L77-66 and L92-71	−/−	H/14.3/82^d^, 90–200	O-glycan structure Deglycosylation	[59]
*Thermoanaerobacterium thermosaccharolyticum*, strain E207-71	*slg* cluster/AY422724	S /~12/83^d^–210	O-glycan structure	[18, 60]
*Thermoanaerobacterium thermosaccharolyticum*, strain D120-70	−/−	S/11/80^d^–170	O-glycan structure Linkage region	[61]
*Thermoanaerobacterium thermosulfurigenes*, strain EM1	−/−	−/−/83^d^–190	SDS- PAGE^b^, PAS^c^ staining Monosaccharide analysis	[62]

Abbreviations: ^a^ O, oblique (p1, p2); S, square (p4); H, hexagonal (p3, p6);  ^b^ SDS-PAGE, sodiumdodecylsulfate polysaccharide gel electrophoresis;  ^c^ PAS, periodic acid-Schiff staining;  ^d^ deglycosylated protein.
